# Predictive value of a self-administered frailty screening questionnaire for the effectiveness of functional rehabilitation evaluated with the locomotor functional independence measure in a geriatric rehabilitation unit: a multicentre cohort study

**DOI:** 10.1186/s12877-024-05605-x

**Published:** 2024-12-19

**Authors:** Jan Chrusciel, Ramatoulaye Ndoye, Biné-Mariam Ndiongue, Marie-Anne Fournier, Fariba Kabirian, Manon Pondjikli, Valentine Dutheillet-de-Lamothe, Gilles Berrut, Yves Rolland, Stéphane Sanchez

**Affiliations:** 1 Department of Public Health, Hôpitaux Champagne Sud, Troyes, France; 2 Research Department, Gérontopôle des Pays de la Loire, Nantes, France; 3 Department of Research and Innovation, Fondation Korian pour le Bien Vieillir, Paris, France; 4 Gérontopôle de Toulouse, CHU de Toulouse, Toulouse, France

**Keywords:** Older people, Functional rehabilitation, Patient-reported outcome measures

## Abstract

**Background:**

Patient Reported Outcome Measures (PROMs) are questionnaires that collect health data directly from the patient, without any intervention from a third party. The aim of rehabilitation units is to restore function. Functional gain can be evaluated with classic scales, such as the locomotor subscale of the Functional Independence Measure. This study aimed to assess the accuracy of a new self-assessment questionnaire pertaining to physical, sensory and cognitive ability (abbreviated SEPCO) for the prediction of functional prognosis in older patients admitted to a rehabilitation unit.

**Methods:**

In this multicentre observational study including patients admitted to 12 rehabilitation centres in France, all included patients completed the SEPCO on admission. Poor response to rehabilitation was defined as relative effectiveness < 40% on the evolution of the locomotor FIM subscale. Components of the questionnaire potentially associated with the outcome of rehabilitation were confirmed for inclusion upon expert review and summed to form an overall score. The final score had five components: the depression score of the HADS, the SOFRESC vision score, the SOFRESC balance score, the stress urinary incontinence subscale of the USP, and the EPICES socio-economic deprivation score. A logistic regression model adjusted for baseline characteristics assessed the performance of the SEPCO score to predict change in functional status, defined by the relative functional gain for the locomotion subscale of the Functional Independence Measure (FIM).

**Results:**

A total of 153 patients (mean age 79.2 ± 8.1 years, 72.5% women) were included. By multivariate analysis, a 5-scale SEPCO score ≥ 1.1 predicted worse functional improvement with an odds ratio (OR) of 2.575, 95% Confidence Interval (CI) 1.081 to 6.133, *p* = 0.03. Sensitivity for this threshold was 67.4% (95% CI 52.0–80.5%), with a specificity of 58.8% (95% CI 46.2–70.6%). Having a SEPCO ≥ 1.1 almost doubled the probability of poor response to rehabilitation (from 27.3 to 52.5%).

**Conclusion:**

The SEPCO score can predict poor functional gain from rehabilitation. Future studies should validate this score on an external cohort. The SEPCO could serve as a complement to the initial clinical evaluation performed by physicians, and assist physicians in setting each patient’s rehabilitation goals.

**Supplementary Information:**

The online version contains supplementary material available at 10.1186/s12877-024-05605-x.

## Introduction

Population aging and medical progress have resulted in increasing numbers of older individuals attending rehabilitation. Identifying the factors that help determine the success of rehabilitation programmes is important to prompt specific interventions aimed at optimizing functional improvement. Patient Reported Outcome Measures (PROMs) are indicators of a patient’s state of health as reported by the patient, without any third-party influence on the response [1]. Although PROMs are widely used in clinical trials, their adoption in routine care is still an emerging phenomenon [2]. PROMs have the potential to detect psychological and/or functional disorders, especially in case of symptoms that may be difficult for an observer to detect (such as fatigue, headache…) or in case of psychological symptoms (such as anxiety or depression). Despite the fact that they include a subjective component, they can also be associated with or predictive of traditional health outcomes like survival [3]. PROMs can be assessed using dedicated tools [4], and may be compared to traditional clinical evaluation methods, for which they are not a replacement. There is an increasing interest in PROMs in the field of rehabilitation [5], and a recent editorial published in the official journal of the American Congress of Rehabilitation Medicine called for an increase in research partnerships between researchers and clinicians regarding this topic [6].

A self-evaluation of possible deficits by the patient could help physicians in identifying the patients’ rehabilitation needs, and ensure that they receive tailored support, with comprehensive, personalized disease management. Frailty is a concept pioneered by Linda Fried [7], and although no consensual definition exists, it can be defined as an alteration in the physiological functions of older people, making them less resistant in case of disease [8]. Older people in a state of frailty are less likely to self-report as healthy, and when compared with their robust counterparts, they probably have lower odds of returning to a normal life with an adequate degree of function after rehabilitation.

However, the creation of new PROMs can be challenging, as their use in clinical practice may be hindered if they are not backed by sufficient evidence. Therefore, we combined a selection of PROMs with known properties to create a comprehensive questionnaire, which we called the “Self-Evaluation of Physical, sensory & COgnitive deficits” (SEPCO).

Unfortunately, some patients do not achieve the expected improvement in motor function at the end of rehabilitation. Although these patients may still have room for improvement after they return home, from the perspective of the rehabilitation centre these cases can be seen as occurrences of negative rehabilitation outcomes.

Being able to predict rehabilitation outcomes could be useful for medical management and in order to set goals for each patient. This prediction would be made possible by creating a score based on the most relevant domains of the initial questionnaire, so that these domains could measure a perceived rehabilitation potential related to the patients’ self-evaluation of their overall health status.

The main objective of this study was therefore to create a score that would be able to identify a subgroup of patients that would struggle to reach locomotor rehabilitation objectives. The main hypothesis was that patients who would identify as the most frail (worst state of health and decreased resilience) on selected domains of the PROM-based questionnaire would achieve lower improvements in locomotion at the end of rehabilitation.

## Methods

### Study design

We performed a prospective, observational, multicentre cohort study, among patients admitted to 12 rehabilitation centres in France between 26 June 2018 and 21 October 2020. The full SEPCO (presented in Table 1) was completed by all patients on admission. Patients were instructed to complete the questionnaire by themselves, but they could seek help if needed. A single investigator in each centre was in charge of obtaining the SEPCO evaluations. Healthcare professionals in each centre also performed evaluations of the Functional Independence Measure (FIM), which was not part of the PROM questionnaire, on admission and at discharge from rehabilitation. The final score was based on domains of the SEPCO that had the highest association with the evolution of the FIM.

A pilot study conducted in January 2017 on 10 patients, with mean age 75 years (minimum 47, maximum 92) had determined that the completion time of the 13-scale SEPCO was approximately 15 to 20 min. Half of the patients (5/10) had required some form of help to finish the questionnaires.

### Study population

The study population comprised older subjects admitted to dedicated rehabilitation centres or hospital-based polyvalent rehabilitation wards. All eligible patients were invited to participate by an investigator in each centre. The healthcare team in each centre was available to answer any questions the patients may have had during the completion of the SEPCO questionnaires. Data were collected on admission and at discharge from rehabilitation. All data were recorded on dedicated case report forms (CRFs).

The inclusion criteria were as follows:

- Provision of written informed consent;
- Male or female patients aged 60 years or older;
- Admitted to one of the 12 participating rehabilitation centres;
- Capable of completing the SEPCO alone (or with minimal help).

The exclusion criteria were as follows:

- Refusal to consent;
- Patients not capable of completing the SEPCO evaluations alone or with minimal help.

### Primary outcome and data recorded

The SEPCO is a set of self-reported evaluation scales chosen to cover 13 clinical domains and enable the detection of physical, sensory or cognitive deficits (Table 1). The initial questionnaires were chosen in order to be as comprehensive as possible, and to cover a variety of systems. Scales to be included in the initial questionnaire were selected by a multidisciplinary team of physicians practicing geriatrics (Pr Yves Rolland), geriatric rehabilitation (Dr Etienne Guarrigues), and hospital managers (Dr Philippe Denormandie). The overall set of questionnaires was expected to be simplified in further versions, according to feedback from professionals and practical relevance. In this study, each patient was asked to complete the full set of SEPCO questionnaires on admission.

**Table 1:** Questionnaires included in the SEPCO

	Domain	Scale included in questionnaire
1	Sleep	Pittsburg Sleep Quality Index (PSQI) [9] (range from 0 to 21, lower is better)
2	Mood	Mini Geriatric Depression Scale [10] (range from 0 to 4, lower is better) and Hospital Anxiety & Depression Scale [11] (both subscales [Anxiety, Depression] range from 0 to 21, lower is better)
3	Pain	Simple Verbal Scale (SVS) (range from 0 to 5, lower is better), and the DN4 (in French: *Douleur Neuropathique-4*) screening tool for neuropathic pain (range from 0 to 10, lower is better) [12, 13]
4	Nutritional status	MNA score BMI category [14] (highest risk of malnutrition if BMI < 19)
5	Cognitive status	Mac Nair memory test [15] (range from 0 to 45, lower is better)
6	Eyesight	Sofresc/AVEC Test, Functional Vision Screening Questionnaire [16] (version with 14 questions; range from 0 to 14, lower is better)
7	Hearing	Sofresc/AVEC Test, screening questionnaire for hearing difficulties [16] (version with 15 questions, range from 0 to 60, lower is better)
8	Balance	Sofresc/AVEC Test [16] (version with three questions, range from 0 to 6, lower is better)
9	Oral health	12-item Geriatric Oral Health Assessment Index [17] (from 0 to 60, higher is better)
10	Fecal continence	Pescatori Questionnaire [18] (from 0 to 24, lower is better)
11	Urinary continence	Urinary Symptom Profile [19] (Stress Incontinence, Overactive bladder and Low stream scores have maximum scores of 24, 9, and 21 respectively, lower is better)
12	Dyspnea	Sadoul dyspnea scale [20] (range from 0 to 9, lower is better)
13	Socio-economic deprivation	EPICES score [21] (range from 0 to 100, lower is better)

The Functional Independence Measure (FIM), which was used to calculate the primary outcome measure (not part of the SEPCO questionnaire), is an instrument that was designed to measure disability, and is widely used to evaluate the response to functional geriatric rehabilitation [22, 23]. It has excellent overall internal consistency, good construct validity, and high test-retest reliability [24]. Its predictive value has been evaluated in patients hospitalized after stroke [25]. Its sensitivity to change is comparable to that of other validated scales [26]. The FIM is composed of 18 items grouped into 6 subscales (self-care, sphincter control, transfers, locomotion, communication and social cognition). The locomotion scale of the FIM was recorded on admission and on discharge. We also recorded the motive for admission, age, sex, date of admission, date of discharge (to calculate length of stay). Motives for admission were classified into 3 categories: category 1 included surgical and post-trauma motives; category 2 included specific medical motives, and category 3 comprised patients with altered general status (see Table S1, Supplementary Material).

### Primary outcome

The primary outcome was evaluated using relative changes in the locomotion scale of the FIM [27] estimated by physicians on admission and discharge. This outcome was evaluated using a measure known as Rehabilitation Effectiveness (RE, also known as Relative Functional Gain or Montebello Rehabilitation Factor Score) [28, 29]. This measure expresses functional gain as a proportion of the maximum gain that could theoretically be achieved. The measure of RE consists in estimating the response to rehabilitation, relative to the maximum possible benefit, using the following equation:

(discharge FIM-admission FIM)/(Maximum possible FIM-Admission FIM) x 100.

Rehabilitation is deemed to be effective if the RE score is ≥ 50% [30]. In our study, we decided to use a lower threshold of 40% for the response to rehabilitation, to account for the capacities of an older population with multiple comorbidities. This threshold was determined before the statistical analysis was conducted. Higher RE thresholds to define rehabilitation success have been used in the literature but predominantly in younger populations [31].

### Statistical methods

#### Sample size

Prior to conducting the study, the proportion of patients with MRFS < 0.4 was evaluated to be 0.53 in robust patients and 0.69 in frail patients (i.e. patients expected to have high scores on the PROM questionnaire). Considering that approximately 25% of patients were expected to be frail, and with an alpha risk of 0.05 and a power of 90%, and accounting for 7% of patients with missing data, 560 patients were to be recruited for the study. Due to slow recruitment and changes in group strategy, the leadership committee decided to close new inclusions after 153 patients were recruited, the decision being effective from 1 August 2020. This decision was taken before any statistical analyses were conducted.

### Statistical analysis

Quantitative data were presented as means ± standard deviations (SD), or medians with the interquartile range (Q1 – Q3) for asymmetric variables, and categorical data as frequencies with their percentages. Average scores were evaluated for each subscale. Predictors of unsuccessful rehabilitation (i.e. RE < 40%) were assessed in bivariate analysis using Student’s *t* test or the Mann-Whitney *U* test (when the variable was asymmetric) for continuous variables, and the χ^2^ test or Fisher’s exact test for categorical variables, as appropriate. Each subscale from the SEPCO evaluation associated with a RE < 40% with a p-value < 0.20 in bivariate analysis was selected as a candidate subscale for the simplified SEPCO score. Candidate subscales for the final score were also validated for inclusion upon expert review of content validity. The subscales were normalized to range between zero and one by dividing by the maximum possible score, and then summed to produce a simplified SEPCO score. The association between the simplified SEPCO score and a RE < 40% was evaluated using a logistic regression model adjusted for variables selected upon expert review: length of stay, patient age, sex, and motive for admission. A sensitivity analysis was conducted with adjustment on the same variables with the exception of length of stay.

Multiple imputation was used for missing data, with m = 200 imputations per model. Sensitivity and specificity of the 5-scale SEPCO score were calculated.

A Confirmatory Factor Analysis (CFA) was conducted on the scales included in the final score [32–34], to assess if they were consistent with a single factor model. The variance of the latent factor was set to one in order to obtain standardized loadings. Study reporting followed the STROBE guidelines [35].

P-values < 0.05 were considered statistically significant. The CFA was performed using the R package *lavaan*. All analyses were performed using R software, version 4.3.3 (The R Foundation for Statistical Computing, Vienna, Austria).

### Ethical considerations

All patients provided written informed consent to participate in the study. The study was approved by the Ethics Committee “Comité de Protection des Personnes Est 1” on 8 March 2018 under the number 2018/21 – ID RCB 2018-A00552-53.

## Results

Based on data provided by the hospital group administration, a total of 6,036 patients were hospitalized in participating hospitals during the study period. The inclusion of patients depended on availability of research staff, patients having sufficient cognitive function, and acceptance to be included by the patient (see reasons for exclusion in Table S2, Supplementary Material).

A total of 153 patients were included over the study period. The RE was missing for 13 patients. A comparison of patient characteristics between participants with RE < 40% and those with RE ≥ 40% is shown in Table 2. Patients with a poor response to rehabilitation had a higher HAD depression score (mean 6.53, Standard Deviation SD 4.58) than patients with a good response to rehabilitation (mean 4.79, SD 3.60, *p* = 0.02). Poor rehabilitation responders also had a higher score on the stress urinary incontinence scale of the USP (1.78 ± SD 2.59) than good responders (mean 0.92 ± SD 2.10, *p* = 0.048). Patients with a poor response to rehabilitation tended to have higher scores on the SOFRESC balance score (mean 2.9, SD 1.9) than patients with a good response to rehabilitation (mean 2.3, SD 1.9, *p* = 0.08). Therefore, for all of the aforementioned scales, the association was in favor of lower rehabilitation success for patients who self-reported as frailest. Body Mass Index (BMI) categories < 19 or ≥ 35 were not associated with rehabilitation success (*p* = 0.69 and 0.59 respectively).

Based on bivariate analysis results, seven scores were preselected to construct the simplified overall SEPCO score: the Mini-GDS score, the depression score of the HADS, the SOFRESC vision score, the SOFRESC balance score, the stress urinary incontinence and overactive bladder subscales of the USP (both included), and the EPICES socio-economic deprivation score. The Mini-GDS and USP overactive bladder subscale were both removed from the final score, as each of them was closely related to another subscale (to the depression score of the HADS and the SOFRESC urinary incontinence subscale, respectively). Therefore, the final score had five components: the depression score of the HADS, the SOFRESC vision score, the SOFRESC balance score, the stress urinary incontinence subscale of the USP, and the EPICES socio-economic deprivation score. After normalization, the simplified SEPCO score could therefore theoretically range from 0 to 5 (each of the five subscales were transformed to continuous variables ranging from 0 to 1 before summation). Patients with poor response to rehabilitation had a mean 5-scale SEPCO score of 1.53 (standard deviation SD 0.70), while patients with successful rehabilitation had a significantly lower score of 1.05 (SD 0.52) (*p* < 0.001). The higher SEPCO score was consistent with higher odds of frailty in patients that fared worse in rehabilitation.

**Table 2:** Comparison of patient characteristics and scores on the SEPCO evaluations according to Rehabilitation Effectiveness (RE) measured with the functional independence measure (FIM) locomotor subscale

	RE < 40% *N* = 50	RE ≥ 40% *N* = 90	*P*-value
Age (years), mean ± SD	79.8 ± 7.9	78.9 ± 8.3	0.52
Sex: male, n (%)	13 (26.5)	25 (28.4)	0.81
Length of stay (days), median [Q1, Q4]	34 [26, 69]	34 [20, 58]	0.43
Motive for admission, n (%)			0.49
Category 1: Surgical/post-trauma	26 (56.5)	55 (66.3)	
Category 2: Medical causes	13 (28.3)	20 (24.1)	
Category 3: Altered general state	7 (15.2)	8 (9.6)	
Five-scale SEPCO score, mean ± SD	1.53 (0.70)	1.05 (0.52)	< 0.001
PSQI score, mean ± SD	9.39 (3.96)	9.10 (3.59)	0.71
Mini-GDS, mean ± SD	1.1 (1.3)	0.7 (1.0)	0.11
HAD Anxiety score, mean ± SD	6.8 (4.3)	6.7 (4.1)	0.87
HAD Depression score, mean ± SD	6.53 (4.58)	4.79 (3.60)	0.02
SVS, mean ± SD	1.2 (1.2)	1.3 (1.3)	0.54
DN4 Score, mean ± SD	1.0 (1.5)	1.3 (1.5)	0.27
Mac Nair score, mean ± SD	10.7 (9.1)	12.4 (9.6)	0.36
SOFRESC Vision score, mean ± SD	2.3 (2.9)	1.7 (1.9)	0.14
SOFRESC balance score, mean ± SD	2.9 (1.9)	2.3 (1.9)	0.08
SOFRESC hearing score, mean ± SD	20.0 (14.7)	17.6 (13.8)	0.38
GOHAI oral health score, mean ± SD	50.5 (7.4)	50.9 (9.4)	0.78
Pescatori score, mean ± SD	0.6 (1.3)	0.5 (1.0)	0.65
USP stress incontinence score, mean ± SD	1.78 (2.59)	0.92 (2.10)	0.05
USP overactive bladder score, mean ± SD	5.2 (4.2)	4.2 (3.9)	0.17
USP low stream score, mean ± SD	0.7 (1.4)	0.7 (1.3)	0.91
Sadoul dyspnea scale, mean ± SD	1.2 (1.4)	1.2 (1.4)	0.90
BMI < 19 kg/m^2^, n (%)	3 (6.8)	4 (4.7)	0.69
Obesity with BMI ≥ 35 kg/m^2^, n (%)	6 (13.6)	9 (10.5)	0.59
EPICES social deprivation, mean ± SD	33.6 (18.3)	28.1 (15.7)	0.10

By multivariate analysis (Table 3), a 5-scale SEPCO score ≥ 1.1 was found to be a significant predictor of unsuccessful rehabilitation (OR, 2.575, 95% CI 1.081–6.133, *p* = 0.03). In other words, patients who self-evaluated as the frailest were least likely to experience a clinically meaningful benefit from rehabilitation. The sensitivity analysis (multivariate analysis without adjustment on length of stay) was consistent with the main analysis (see Table S3, Supplementary Material).

**Table 3:** Multivariate analysis by logistic regression estimating the probability of unsuccessful rehabilitation (RE < 40%) - pooled estimates from multiple imputation for missing data (m = 200 imputations)

	Adjusted Odds Ratio^a^ (aOR)	95% confidence interval, lower bound	95% confidence interval, upper bound	*P*-value
Age (continuous variable), OR per additional year	0.998	0.95	1.049	0.94
Sex: male (Ref.: female)	1.168	0.488	2.799	0.73
Length of stay (continuous variable), OR per additional day	1.005	0.994	1.016	0.39
Motive for admission				0.56
Category 1: Surgical/post-trauma	1 (Ref.)			
Category 2: Medical causes	1.414	0.567	3.528	
Category 3: Altered general state	1.86	0.5	6.914	
Five-scale SEPCO score ≥ 1.1 (Ref.: < 1.1)	2.575	1.081	6.133	0.03

^a^Adjusted on: age, sex, length of stay, motive for admission, SEPCO score ≥ 1.1. Ref.: reference category. Area Under the Curve (AUC): 0.658; Nagelkerke pseudo-R^2^: 0.10. RE: Rehabilitation Effectiveness, measured with the Functional Independence Measure locomotor subscale

A simplified SEPCO score ≥ 1.1 identified patients who were less likely to respond to rehabilitation with a sensitivity of 67.4% (95% CI 52.0–80.5%) and a specificity of 58.8% (95% CI 46.2–70.6%). Having a 5-score SEPCO ≥ 1.1 almost doubled the probability of poor response to rehabilitation (from 27.3 to 52.5%).

The CFA conducted on the final 5-scale SEPCO showed good fit indices but relatively weak standardized loadings for a single factor model (see Figure S1 and Table S4, Supplementary Material).

## Discussion

This study shows that self-reported sensory and cognitive function using the simplified SEPCO score (constituted by the SOFRESC balance and vision subscales, the HAD depression scale, the USP urinary stress scale and the EPICES socioeconomic scale) was predictive of lower rehabilitation effectiveness, defined as a RE < 40% measured with the locomotor scale of the Functional Improvement Measure, in older adults admitted to rehabilitation. By multivariate analysis, the length of stay in rehabilitation was not significantly associated with the success of the rehabilitation program. The CFA suggests a relatively multidimensional structure, which is not uncommon for scores purporting to measure frailty, which can model several largely independent systems, each having a distinct role in predicting adverse outcomes [36].

The SEPCO presents some similarities to the WHO Integrated Care for Older People (ICOPE) strategy, and could be described as a French adaptation of this WHO framework for the management of the frailest elderly [37]. Indeed, the SEPCO evaluations enable rapid referral of patients to specialized care when deficits are identified, with the added advantage of being a PROM, thereby involving the patient in their diagnostic and management process from the outset [38]. As new generations of older adults become increasingly at ease with information technologies, the various questionnaires in the SEPCO could be administered via applications, like those used in the ICOPE project [39]. A questionnaire covering more domains than SEPCO exists in Korea (Korean Frailty Index for Primary Care) [40], but it requires the presence of a physician to be administered, and thus is not strictly speaking a self-report tool. In a study from France, the ICOPE Step 1 screening tool was adapted and applied to 759 patients participating in the Multidomain Alzheimer Preventive Trial (MAPT) [41, 42]. The authors reported that using this adapted tool, 90% of older adults had one or more conditions associated with declining capacity. However, to the best of our knowledge, few studies have been conducted using an evaluation as broad as the SEPCO in the context of functional rehabilitation. Our evaluation covered indicators of continence, an intrinsic capacity that is notably absent from the ICOPE Step 1 [38]. While continence appeared mostly preserved in older persons in a recent ICOPE study (96.5%) [43], loss of continence was found to be associated with less effective rehabilitation in our study.

The variables found to be predictive of rehabilitation outcomes in our study are largely in line with those reported in the literature. Urinary incontinence has previously been identified as a predictor of RE score, in patients with stroke [28]. The mechanisms leading to the reduced efficacy of rehabilitation in these patients could involve reduced capacity for self-care, and fewer social interactions [44]. In line with the findings of a previous study [45], BMI was not associated with rehabilitation efficiency, although few of our patients had high BMIs and therefore our study may have lacked power for this purpose. We used a high threshold of 35 kg/m^2^ to identify obese patients, as some studies has suggested that the optimal BMI for older people may be higher than for the young (who are considered obese for BMI ≥ 25 kg/m^2^) [46, 47]. A BMI > 30 was nonetheless associated with higher costs in the study by Vincent [45]; and this is a point that should not be overlooked.

### Study strengths and limitations

The broad inclusion criteria of this study enabled us to include a general population, meaning that our results can be extrapolated to all patients admitted to rehabilitation programmes, providing the patients are autonomous enough to complete all the evaluations satisfactorily. The self-reported nature of the evaluations is advantageous, in that it gets the patient actively involved in their healthcare pathway from the outset. However, as with all self-report tools, there may be some question as to the reliability of the patients’ answers. The association observed between perceived frailty and the efficacy of functional rehabilitation nonetheless confirms that in addition to being dependent on objective components of disease, performance during rehabilitation is conditioned by the patient’s perceived frailty, as reported in the questionnaire, despite its attendant subjectivity. Our questionnaire covered a wide range of medical problems and diseases, thus reducing the potential for residual confounding. Nonetheless, all domains could not be assessed, and the score does not contain a detailed physical function assessment. Numerous patients were excluded from participation in the study due to health issues or the unavailability of staff to assist with completion of the questionnaire. The decision to include a patient was based on the physicians’ judgement rather than on a standardized cognitive ability test. Future studies should evaluate if the short form of the SEPCO is easier to complete than the 13-scale version, and whether most patients can finish the abridged questionnaires with minimal assistance. The number of patients recruited was lower than initially expected, which meant that it was difficult to include more strata in the classification of admission causes while retaining adequate representativity within each class, or to separate the data in a training set and a validation set to estimate out-of-sample predictive accuracy. A larger sample could also increase the representativity of included patients. These issues could be addressed in further studies. The FIM motor function items have been criticized for not being unidimensional (although this claim was debated) [48, 49], and future studies may need to rely on other validation instruments. Finally, the structure of the SEPCO appears to have multiple dimensions, which complicates the interpretation of the underlying latent construct. This complexity is often seen in instruments that attempt to measure frailty, a concept that may involve alterations on multiple systems interacting with the patients’ environment in a complex manner. However, this should not by itself discourage use of the SEPCO, as scores constructed with a primary aim of prediction can include items that cover a variety of domains or systems [36]. Future iterations of the tool should be designed with simplicity in mind and a neutral formulation of questions, to avoid negative reactions by the patient. Finally, although the 5-scale SEPCO can give indications regarding the probability of poor response to rehabilitation, in most cases it is not sufficient for this purpose and should always be used in conjunction with other evaluation means.

## Conclusions

We showed that PROM results, represented by the SEPCO questionnaire, were associated with the response to functional rehabilitation in a population of older adults admitted to rehabilitation. The predictive power and acceptability of the abridged five-scale SEPCO score should be further evaluated on a separate cohort in a prospective study. The SEPCO questionnaire could then be administered to patients entering rehabilitation in order to tailor the programme to their individual needs. The SEPCO score could be a complement to the physician’s initial evaluation, without replacing it.

## Electronic supplementary material

Below is the link to the electronic supplementary material.

Supplementary Material 1

## Data Availability

The datasets analysed during the current study are available from the corresponding author on reasonable request.

## References

[1] Deshpande PR, Rajan S, Sudeepthi BL, Abdul Nazir CP. Patient-reported outcomes: a new era in clinical research. Perspect Clin Res. 2011;2:137–44. 22145124 10.4103/2229-3485.86879 PMC3227331

[2] Benson T. Why it is hard to use PROMs and PREMs in routine health and care. BMJ Open Qual. 2023;12:e002516. 38135303 10.1136/bmjoq-2023-002516 PMC10749067

[3] Ediebah DE, Coens C, Zikos E, Quinten C, Ringash J, King MT, et al. Does change in health-related quality of life score predict survival? Analysis of EORTC 08975 lung cancer trial. Br J Cancer. 2014;110:2427–33. 24743709 10.1038/bjc.2014.208 PMC4021536

[4] Weldring T, Smith SMS. Patient-reported outcomes (PROs) and patient-reported outcome measures (PROMs). Health Serv Insights. 2013;6:61–8. 25114561 10.4137/HSI.S11093 PMC4089835

[5] Müller M, Brunssen J, Messingschlager M. Patient reported outcomes (PROs) - a Tool for strengthening patient involvement and measuring outcome in Orthopaedic Outpatient Rehabilitation. Z Orthopadie Unfallchirurgie. 2022;160:84–92. 10.1055/a-1241-4897 33045757

[6] Keeney T, Kumar A, Erler KS, Karmarkar AM. Making the case for patient-reported outcome measures in Big-Data Rehabilitation Research: implications for optimizing patient-centered care. Arch Phys Med Rehabil. 2022;103:S140–5. 33548207 10.1016/j.apmr.2020.12.028 PMC10227674

[7] Fried LP, Tangen CM, Walston J, Newman AB, Hirsch C, Gottdiener J, et al. Frailty in older adults: evidence for a phenotype. J Gerontol Biol Sci Med Sci. 2001;56:M146–57. 10.1093/gerona/56.3.m146 11253156

[8] Rockwood K, Mitnitski A. Frailty defined by deficit accumulation and geriatric medicine defined by frailty. Clin Geriatr Med. 2011;27:17–26. 21093719 10.1016/j.cger.2010.08.008

[9] Zhong Q-Y, Gelaye B, Sánchez SE, Williams MA. Psychometric Properties of the Pittsburgh Sleep Quality Index (PSQI) in a cohort of Peruvian pregnant women. J Clin Sleep Med JCSM off Publ Am Acad Sleep Med. 2015;11:869–77. 10.5664/jcsm.4936 PMC4513264 25845902

[10] Clement JP, Fray E, Paycin S, Leger JM, Therme JF, Dumont D. Detection of depression in elderly hospitalized patients in emergency wards in France using the CES-D and the mini-GDS: preliminary experiences. Int J Geriatr Psychiatry. 1999;14:373–8. 10389041 10.1002/(sici)1099-1166(199905)14:5<373::aid-gps923>3.0.co;2-s

[11] Zigmond AS, Snaith RP. The hospital anxiety and depression scale. Acta Psychiatr Scand. 1983;67:361–70. 6880820 10.1111/j.1600-0447.1983.tb09716.x

[12] Haefeli M, Elfering A. Pain assessment. Eur Spine J. 2006;15(Suppl 1):S17–24. 16320034 10.1007/s00586-005-1044-x PMC3454549

[13] Mulvey MR, Boland EG, Bouhassira D, Freynhagen R, Hardy J, Hjermstad MJ, et al. Neuropathic pain in cancer: systematic review, performance of screening tools and analysis of symptom profiles. Br J Anaesth. 2017;119:765–74. 29121284 10.1093/bja/aex175

[14] Guigoz Y, Vellas B, Garry PJ. Assessing the nutritional status of the elderly: the Mini Nutritional Assessment as part of the geriatric evaluation. Nutr Rev. 1996;54(1 Pt 2):S59–65. 8919685 10.1111/j.1753-4887.1996.tb03793.x

[15] Dardenne S, Delrieu J, Sourdet S, Cantet C, Andrieu S, Mathiex-Fortunet H, et al. Memory complaints and Cognitive decline: data from the GUIDAGE Study. J Alzheimers Dis. 2017;60:1567–78. 28984580 10.3233/JAD-170229

[16] SOFRESC. Kit de repérage des fragilités sensori-cognitives. 2015. Available on: https://www.sofresc.com/test-avec/

[17] Tubert-Jeannin S, Riordan PJ, Morel-Papernot A, Porcheray S, Saby-Collet S. Validation of an oral health quality of life index (GOHAI) in France. Community Dent Oral Epidemiol. 2003;31:275–84. 12846850 10.1034/j.1600-0528.2003.t01-1-00006.x

[18] Pescatori M, Anastasio G, Bottini C, Mentasti A. New grading and scoring for anal incontinence. Evaluation of 335 patients. Dis Colon Rectum. 1992;35:482–7. 1568401 10.1007/BF02049407

[19] Haab F, Richard F, Amarenco G, Coloby P, Arnould B, Benmedjahed K, et al. Comprehensive evaluation of bladder and urethral dysfunction symptoms: development and psychometric validation of the urinary Symptom Profile (USP) questionnaire. Urology. 2008;71:646–56. 18313122 10.1016/j.urology.2007.11.100

[20] Sadoul P. Evaluation Du déficit fonctionnel respiratoire. Bull Eur Physiopathol Respir. 1983;19:3–6. 6850145

[21] Sass C, Guéguen R, Moulin JJ, Abric L, Dauphinot V, Dupré C, et al. [Comparison of the individual deprivation index of the French Health Examination Centres and the administrative definition of deprivation]. Sante Publique Vandoeuvre–Nancy Fr. 2006;18:513–22. 10.3917/spub.064.0513 17294755

[22] Muir-Hunter SW, Fat GL, Mackenzie R, Wells J, Montero-Odasso M. Defining Rehabilitation Success in older adults with dementia–results from an Inpatient Geriatric Rehabilitation Unit. J Nutr Health Aging. 2016;20:439–45.26999245 10.1007/s12603-015-0585-x

[23] Glenny C, Stolee P. Comparing the functional independence measure and the interRAI/MDS for use in the functional assessment of older adults: a review of the literature. BMC Geriatr. 2009;9:52. 19943969 10.1186/1471-2318-9-52 PMC2795323

[24] Dodds TA, Martin DP, Stolov WC, Deyo RA. A validation of the functional independence measurement and its performance among rehabilitation inpatients. Arch Phys Med Rehabil. 1993;74:531–6. 8489365 10.1016/0003-9993(93)90119-u

[25] Timbeck RJ, Spaulding SJ. Ability of the functional independence Measure^™^ to Predict Rehabilitation outcomes after Stroke: a review of the literature. Phys Occup Ther Geriatr. 2004;22:63–76.

[26] van der Putten JJMF, Hobart JC, Freeman JA, Thompson AJ. Measuring change in disability after inpatient rehabilitation: comparison of the responsiveness of the Barthel Index and the functional independence measure. J Neurol Neurosurg Psychiatry. 1999;66:480–4. 10201420 10.1136/jnnp.66.4.480 PMC1736299

[27] Adunsky A, Lusky A, Arad M, Heruti RJ. A comparative study of rehabilitation outcomes of elderly hip fracture patients: the advantage of a comprehensive orthogeriatric approach. J Gerontol Biol Sci Med Sci. 2003;58:542–7. 10.1093/gerona/58.6.m542 12807926

[28] Koh GC-H, Chen CH, Petrella R, Thind A. Rehabilitation impact indices and their independent predictors: a systematic review. BMJ Open. 2013;3:e003483. 24068767 10.1136/bmjopen-2013-003483 PMC3787469

[29] Rolland Y, Pillard F, Lauwers-Cances V, Busquère F, Vellas B, Lafont C. Rehabilitation outcome of elderly patients with hip fracture and cognitive impairment. Disabil Rehabil. 2004;26:425–31. 15204479 10.1080/09638280410001663148

[30] Milman R, Zikrin E, Shacham D, Freud T, Press Y. Handgrip Strength as a predictor of successful Rehabilitation after hip fracture in patients 65 years of Age and Above. Clin Interv Aging. 2022;17:1307–17. 36072307 10.2147/CIA.S374366 PMC9441578

[31] Franceschini M, Massimiani MP, Paravati S, Agosti M. Return to work: a cut-off of FIM Gain with Montebello Rehabilitation Factor Score in Order to identify predictive factors in subjects with acquired Brain Injury. PLoS ONE. 2016;11. 10.1371/journal.pone.0165165 PMC5079591 27780215

[32] Hu L, Bentler PM. Cutoff criteria for fit indexes in covariance structure analysis: conventional criteria versus new alternatives. Struct Equ Model. 1999;6:1–55.

[33] Schumacker RE, Lomax RG. A beginner’s guide to structural equation modeling. 2nd ed. New York: Psychology; 2004.

[34] Awang Z. A handbook on structural equation modeling using AMOS. 1st ed. Shah Alam, Malaysia: Universiti Technologi MARA; 2012.

[35] Vandenbroucke J, von Elm E, Altman D, Gøtzsche P, Mulrowe C, Pocock S, et al. The strengthening the reporting of Observational studies in Epidemiology (STROBE) statement: guidelines for reporting observational studies. Ann Intern Med. 2007;147:W163–94. 17938389 10.7326/0003-4819-147-8-200710160-00010-w1

[36] Gilbert T, Neuburger J, Kraindler J, Keeble E, Smith P, Ariti C, et al. Development and validation of a hospital frailty risk score focusing on older people in acute care settings using electronic hospital records: an observational study. Lancet. 2018;391:1775–82. 29706364 10.1016/S0140-6736(18)30668-8 PMC5946808

[37] Tavassoli N, Piau A, Berbon C, De Kerimel J, Lafont C, De Souto Barreto P, et al. Framework implementation of the INSPIRE ICOPE-CARE program in collaboration with the World Health Organization (WHO) in the Occitania Region. J Frailty Aging. 2021;10:103–9. 33575698 10.14283/jfa.2020.26

[38] World Health Organization. Guidance on person-centred assessment and pathways in primary care. Handbook. Geneva: World Health Organization; 2019. p. 87. https://apps.who.int/iris/bitstream/handle/10665/326843/WHOFWC-ALC-19.1-eng.pdf?sequence=17&isAllowed=y.

[39] Sanchez-Rodriguez D, Annweiler C, Gillain S, Vellas B. Implementation of the Integrated Care of Older people (ICOPE) app in primary care: New technologies in Geriatric Care during Quarantine of COVID-19 and Beyond. J Frailty Aging. 2021;10:139–40. 33575702 10.14283/jfa.2020.24 PMC7195618

[40] Won CW, Ha E, Jeong E, Kim M, Park J, Baek JE, et al. World Health Organization Integrated Care for older people (ICOPE) and the Integrated Care of older patients with Frailty in Primary Care (ICOOP_Frail) study in Korea. Ann Geriatr Med Res. 2021;25:10–6. 33794585 10.4235/agmr.21.0025 PMC8024169

[41] González-Bautista E, De Souto Barreto P, Virecoulon Giudici K, Andrieu S, Rolland Y, Vellas B, et al. Frequency of conditions Associated with declines in intrinsic capacity according to a Screening Tool in the context of Integrated Care for older people. J Frailty Aging. 2021;10:94–102. 33575697 10.14283/jfa.2020.42

[42] Sum G, Lau LK, Jabbar KA, Lun P, George PP, Munro YL, et al. The World Health Organization (WHO) Integrated Care for older people (ICOPE) Framework: a narrative review on its Adoption Worldwide and lessons Learnt. Int J Environ Res Public Health. 2023;20:154. 10.3390/ijerph20010154 PMC9819593 36612480

[43] Prince MJ, Acosta D, Guerra M, Huang Y, Jacob KS, Jimenez-Velazquez IZ, et al. Intrinsic capacity and its associations with incident dependence and mortality in 10/66 Dementia Research Group studies in Latin America, India, and China: a population-based cohort study. PLOS Med. 2021;18:e1003097. 34520466 10.1371/journal.pmed.1003097 PMC8439485

[44] Leung AYM, Su JJ, Lee ESH, Fung JTS, Molassiotis A. Intrinsic capacity of older people in the community using WHO Integrated Care for older people (ICOPE) framework: a cross-sectional study. BMC Geriatr. 2022;22:304. 35395736 10.1186/s12877-022-02980-1 PMC8993034

[45] Vincent HK, Vincent KR, Lee LW, Alfano AP. Effect of obesity on inpatient rehabilitation outcomes following total knee arthroplasty. Clin Rehabil. 2007;21:182–90. 17264112 10.1177/0269215506069245

[46] Kıskaç M, Soysal P, Smith L, Capar E, Zorlu M. What is the optimal body Mass Index Range for older adults? Ann Geriatr Med Res. 2022;26:49–57. 35368193 10.4235/agmr.22.0012 PMC8984168

[47] Winter JE, MacInnis RJ, Wattanapenpaiboon N, Nowson CA. BMI and all-cause mortality in older adults: a meta-analysis. Am J Clin Nutr. 2014;99:875–90. 24452240 10.3945/ajcn.113.068122

[48] Dickson HG, Köhler F. The multi-dimensionality of the FIM motor items precludes an interval scaling using Rasch analysis. Scand J Rehabil Med. 1996;28:159–62. 8885038

[49] Wright BD, Linacre JM, Smith RM, Heinemann AW, Granger CV. FIM measurement properties and Rasch model details. Scand J Rehabil Med. 1997;29:267–72. 9428061

