# Supplementary material for: Predictive value of a self-administered frailty screening questionnaire for the effectiveness of functional rehabilitation evaluated with the locomotor functional independence measure in a geriatric rehabilitation unit: a multicentre cohort study
Source: BMC Geriatr. 2024 Dec 19;24:1013. doi: 10.1186/s12877-024-05605-x (PMC11656562; doi:10.1186/s12877-024-05605-x)
Supplement: Supplementary file 1 — Supplementary Material 1 [file 12877_2024_5605_MOESM1_ESM.pdf]

Supplementary Material 1

Table S1: Motives for admission - details of the three patient categories

**Category 1**<br><br>**Surgery/post-trauma**	**Category 2**<br><br>**Medical conditions**	**Category 3**<br><br>**Altered general status**
Fracture of the humerus	Pancreatic adenocarcinoma	To provide relief for family caregivers
Fracture of the ankle	Stroke	Falls (without specific cause identified)
Fracture of the femoral neck	Cancer	Malaise
Removal of osteosynthesis devices	Infection	Loss of autonomy
Neurosurgery	Ischemic colitis	Absence of main caregiver
Mastectomy	Malnutrition	Altered general status
Colectomy	Epilepsy	
Cholecystectomy	Right pontine hemorrhage	
Knee prosthesis	Acute cor pulmonale	
Hip prosthesis	Pneumonia	
Shoulder prosthesis	Deep vein thrombosis	
Arthrodesis	Lower back pain	
Other surgery	Hyponatremia	
	Heart failure	
	Cardio-respiratory disorders	
	Febrile neutropenia	

Table S2: Reasons provided by participating hospitals for not including patients

Centre	Number of inclusions	Estimated number of admissions over study period	Reasons for exclusion
Centre 1	11	312	Staff not available and/or patients not willing to participate and/or not meeting inclusion criteria
Centre 2	21	648	Patient refusal.<br>Patients not inclined to provide consent.<br>Study inclusion leaflet not understood by patients.
Centre 3	3	693	Reasons not provided<br>(low availability of research resources)
Centre 4	17	990	Patients find that the questionnaire contains sensitive information.<br>Few patients are able to complete questionnaire due to old age and dependency.
Centre 5	8	Not available	Most patients were not able to complete the questionnaire without help.<br>Patients are dependent.
Centre 6	24	847	Refusal to participate.<br>Patient state not compatible with completion of questionnaire.
Centre 7	6	420	Refusal to complete questionnaire/research staff not available
Centre 8	4	450	Less than 10% of patients met inclusion criteria. Patients were too old and had low cognitive function (mean age 86 years).<br>Patients were dependent.<br>End of life context.<br>Medical comorbidities not compatible with rehabilitation.
Centre 9	8	580	Dependence.<br>End of life context.<br>Cognitive ability does not allow for completion of questionnaire.
Centre 10	22	500	Old age and cognitive decline.<br>End of life.<br>Altered general state due to comorbidity.<br>Patients were reticent to provide the requested information.
Centre 11	7	260	Patient refusal.<br>Numerous patients had cognitive issues (mean age 75 years).<br>State of dependency.
Centre 12	22	336	Older patients were very dependent and not able to complete questionnaire due to cognitive alterations.<br>End of life context.<br>Some of the patients were transferred.<br>Patients did not understand study inclusion leaflet.

Table S3: Multivariate analysis by logistic regression predicting the probability of unsuccessful rehabilitation (RE <40%), without adjustment on length of stay - pooled estimates from multiple imputation for missing data (m=200 imputations).

	Adjusted Odds Ratio[a] (aOR)	95% confidence interval, lower bound	95% confidence interval, upper bound	P-value
Age (continuous variable), OR per additional year	0,998	0,95	1,048	0.93
Sex: male (Ref.: female)	1,171	0,491	2,793	0.72
Motive for admission				0.62
Category 1: Surgical/post-trauma	1 (Ref.)			
Category 2: Medical causes	1,325	0,539	3,254	
Category 3: Altered general state	1,786	0,490	6,514	
Five-scale SEPCO score ≥ 1.1 (Ref.: score < 1.1)	2,568	1,073	6,148	0.03

[a]Adjusted on: age, sex, motive for admission, SEPCO score ≥ 1.1. Ref.: reference category. Area Under the Curve (AUC): 0.649; Nagelkerke pseudo-R^2: 0.09. RE: Rehabilitation Effectiveness (measured with the Functional Independence Measure locomotor subscale)

Figure S1: Confirmatory Factor Analysis (CFA) of the final SEPCO score

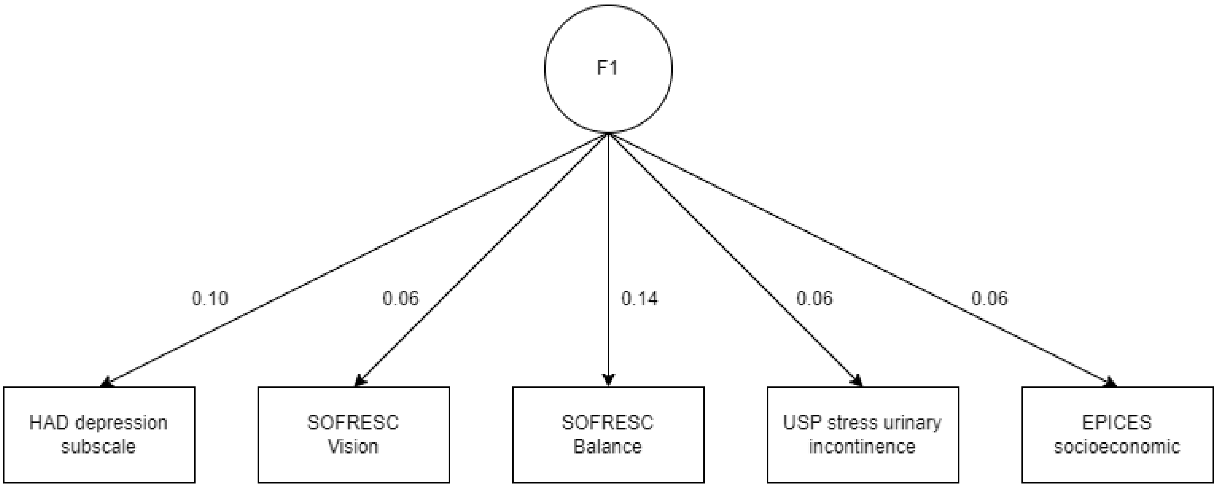

Coefficients represent standardized loadings. The CFA model had adequate fit statistics: a Comparative Fit Index (CFI) of 0.983 (satisfactory fit when ≥ 0.96), a Tucker-Lewis Index (TLI) of 0.965 (satisfactory fit if ≥ 0.95), Chi-2 : p = 0.38 (test statistic 5.308, Degrees of Freedom (DF) = 5; not rejecting the null hypothesis indicates good fit when sample size is moderate), Root Mean Square Error of Approximation (RMSEA): 0.02 (closer to zero indicates a good fit, values < 0.08 are satisfactory). Although the threshold may vary according to context, factor loadings > 0.5 are considered strongly related to the latent variable.

Table S4: Confirmatory Factor Analysis (CFA) of the SEPCO score: factor loadings

	Standardized loading	P-value
**HAD depression subscale**	0.10	0.002
**SOFRESC Vision**	0.06	0.003
**SOFRESC Balance**	0.14	0.001
**USP stress urinary incontinence**	0.062	0.051
**EPICES socioeconomic evaluation**	0.060	0.01
